# Higher prevalence of obesity and overweight without an adverse metabolic profile in girls with central precocious puberty compared to girls with early puberty, regardless of GnRH analogue treatment

**DOI:** 10.1186/1687-9856-2014-5

**Published:** 2014-04-17

**Authors:** Ana Colmenares, Peter Gunczler, Roberto Lanes

**Affiliations:** 1 Department of Pediatrics, Hospital Dr. Patrocinio Peñuela-IVSS, San Cristobal, Táchira 5001, Venezuela; 2 Pediatric Endocrine Unit, Hospital de Clínicas Caracas, Caracas, Venezuela

**Keywords:** Central precocious puberty, Early puberty, GnRHa, BMI, Overweight and obesity rates, Glucose, Lipids

## Abstract

**Objectives:**

1. To determine BMI, obesity/overweight rates, glucose and lipids at baseline, during GnRHa treatment and shortly after therapy discontinuation in female children with CPP and EP. 2. To compare this response to that seen in a similar group of untreated patients.

**Methods:**

A retrospective analysis of 71 children with either CPP (n = 37) or EP (n = 34) was undertaken. Forty three were treated with a GnRHa for at least 2 years, while 28 were followed without treatment.

**Results:**

At the time of diagnosis, a higher BMI (z-score of 1.1 ± 0.8 vs. 0.6 ± 0.7, p = 0.004) and a higher prevalence of obesity/overweight (72.9 vs. 35.3%, p = 0.001) was observed in subjects with CPP when compared to those with EP. Children with EP had higher fasting glucose and total cholesterol than those with CPP. BMI z-score, obesity/overweight rates, fasting glucose and lipids did not change significantly in girls with CPP or EP during 3 yrs of follow up, regardless of treatment. Weight z-scores were higher at 3 years in treated than in untreated girls with CPP (p = 0.02), while it was higher in untreated than in GnRHa-treated patients with EP at baseline, 1, 2 and 3 years (p = 0.007, p = 0.002, p = 0.02 and p = 0.04, respectively) and remained so shortly after stopping therapy (p = 0.03).

**Conclusions:**

There is a high prevalence of obesity/overweight in girls with CPP and EP at diagnosis. However, this risk is greater in CPP than in EP girls. BMI, Obesity/overweight rates, fasting glucose and lipids remained stable in CPP and EP girls regardless of therapy. Weight z-scores were found to be higher in treated CPP girls and in untreated girls with EP.

## Introduction

Central precocious puberty (CPP) and early puberty (EP) may be associated with an increased body mass index (BMI), adiposity, and with an increased prevalence of obesity before, during and after discontinuing GnRH analog (GnRHa) treatment when compared with age- and sex-matched reference values of the same population [1-4]. Until 2012, around 20 studies had addressed the BMI outcome and the prevalence of obesity in children with CPP or EP treated with a GnRHa [1-3,5-17]. Of these only two studies analyzed patients with EP separately [18,19]. Additionally only two previous publications reported on the evolution of glucose and lipids during GnRHa therapy [4,20]. The consensus statement on the use of GnRHa in children [21] highlighted the need for studies regarding body composition, fat distribution or metabolic syndrome in this population.

Data in the literature concerning the influence of GnRHa therapy on the evolution of BMI and the rate of obesity-overweight in children with CPP and EP remain controversial. In some studies, obesity in patients with CPP seems to be unrelated to GnRHa administration [1,6] and girls treated in childhood with a GnRHa have been found to have a normal BMI and body composition in early adulthood [17]. However in other reports a positive correlation between BMI before GnRHa administration and BMI after this treatment has been shown [7,22] and an increase in BMI, total body fat, and in insulin resistance has been described in some GnRHa-treated CPP subjects [4,15,23]. Only one publication documented a reduction of BMI z-score and of the obesity rate in girls with CPP during GnRHa treatment [11].

In this unicenter study we assessed BMI, rate of obesity and overweight, as well as fasting glucose and lipid profiles at the time of diagnosis, during at least 2 years of GnRHa therapy and shortly after treatment discontinuation in a group of 71 girls affected by CPP or by EP. We compared results at these time periods between CPP and EP patients with a similar group of untreated patients.

### Subjects and methods

The medical records of 130 girls with a diagnosis of early or precocious puberty from the Pediatric Endocrine Unit of the Hospital de Clínicas Caracas were screened. Of these we selected only those records (n = 71) that met the following inclusion criteria: 1. Confirmed CPP or EP: a) onset of breast development (stage B2 or above according to Tanner) before a chronological age of 9 yrs, b) pubertal LH response (>7 IU/liter) to a GnRH stimulation test, c) advancement of bone age over chronological age by at least 1 yr, d) pubertal uterine and ovarian volume at pelvic ultrasonography [24]. 2. No evidence of hypothalamic-pituitary organic lesions following magnetic resonance imaging. 3. Absence of other conditions that might affect the onset of puberty and the BMI (e.g. GH deficiency, congenital adrenal hyperplasia, hypothyroidism). 4. Regular follow up during at least 2 years. 5. Measurement of lipid and glucose levels once a year during at least 2 years of follow up. 6. Compliance with the GnRHa treatment during the period of follow up. 7. Confirmed gonadotropin suppression throughout the period of GnRHa administration.

According to criteria previously established, we excluded all patients with isolated thelarche, non progressive forms of puberty, normal puberty, non confirmed laboratory tests suggestive of advanced puberty at diagnosis, incomplete medical records, irregular follow up, absence of lipid and glucose measurements at baseline or at follow, less than 2 years of follow up and no treatment compliance.

Patients were diagnosed with CPP (n = 37) if clinically and biochemically confirmed puberty presented at less than 8 yrs of age and they were diagnosed with EP (n = 34) if puberty was confirmed between the ages of 8–9 yrs. Based on the therapeutic policy of our Pediatric Endocrine Unit at that time, gonadotropin-suppressive therapy was offered to all the girls after an observation period of 1 to 3 months in order to rule out transient or slowly progressive forms of puberty, which do not require treatment. Therapy was offered on the basis of either: 1. A diagnosis of a rapidly progressive form of CPP, regardless of stature, 2. A rapidly progressive form of EP associated with emotional difficulties or a low predict adult height. Of the CPP girls, 29 accepted treatment, 5 had a slowly progressive form of CPP and were therefore not treated and 3 refused therapy. Of the EP girls, 20 were followed without therapy, while 14 were treated. Those who had a slowly progressive form of CPP and EP or refused therapy were included in the study as controls.

GnRHa treatment consisted of depot-triptorelin, 3.75 mg im every 28 days. During treatment, gonadotropin suppression was regularly confirmed clinically and by pre-pubertal levels of LH following acute iv LHRH stimulation (LH <2 mIU/ml). Therapy was discontinued at a bone age of 11.5 to 12.5 yrs. The duration of GnRHa-therapy was 2–4 yrs.

Patients with an elevated BMI at every evaluation were recommended to increase physical activity and to improve nutritional habits (to eat more fruits, grains and vegetables and to decrease their caloric intake). The number of patients in each group that were recommended to diet and to increase physical activity was not significantly different (CPP: 79.3 vs. 50%, p = 0.17 and EP: 29 vs. 40%, p = 0.71, for treated vs. untreated patients respectively).

## Methods

A retrospective analysis of the medical records of these patients was undertaken. All patients had been evaluated every 3–4 months by the same pediatric endocrinologist. Body weight was measured with an electronic scale to the nearest 0.1 kg. Height was measured with a Harpender stadiometer to the nearest millimeter. BMI was calculated as weight (kilograms)/height (meter square) and was expressed as Z-score (z-scores) for chronological age according to the CDC reference range [25]. Using Venezuelan BMI percentile cut-off values, we defined overweight as above the 90th percentile and obesity as above the 97th percentile [26]. Centile curves were drawn so that at age 18 years they passed through the widely used cut off points of 25 and 30 kg/m(2) for adult overweight and obesity.

Laboratory evaluation included measurement of LH, FSH, estradiol, fasting glucose and lipid levels (total cholesterol, high density lipoprotein cholesterol (HDL-C), and triglycerides). According to hospital policy blood samples were obtained early in the morning in the fasting state in all patients and with the proper parental approval. Serum LH, FSH, and estradiol levels were measured by chemoluminescence in an Immulite autoanalyzer using reagents from Diagnostic Product Corporation (DPC, Los Angeles, USA). The intra- and interassay coefficients of variation (CVs) were 5.7-6.1% and 5.3-6.5% for LH and FSH and 6.3%-6.4% for estradiol, respectively. Serum glucose, total cholesterol, HDL-C and triglycerides where measured by enzymatic methods with a Technicon autoanalyzer (Boehringer Mannheim Diagnostics). LDL-C was estimated using the Friedewald formula (LDL-C = TC – Tg/5 + HDL-C) [27]. The triglyceride/HDL-C ratio was calculated [28]. All assays were performed in the clinical biochemistry laboratory of the Hospital. Total cholesterol and triglyceride levels were compared to equivalent Venezuelan reference data [26].

The data shown at baseline corresponds to the start of treatment in GnRHa treated patients and the time of confirmed diagnosis in non-treated patients. This means that all patients had the same short period of follow up between the first visit and the inclusion into the study. We did not observe any significant difference in the BMI of our patients during this short period of observation. In the analysis we also include the clinical data from 9 patients shortly after discontinuation of GnRHa therapy (6–12 months; mean of 9 ± 3 months). These patients had previously received GnRHa treatment for at least 2 years and therefore, all of them were included in the 2 year analysis of the GnRHa treated group.

The institutional review board of the hospital approved the study, and informed consent for inclusion into the study was obtained from all adolescents and their parents.

### Statistical analysis

All values are expressed as mean ± SDS unless otherwise stated. Differences in categorical variables between the groups were tested by the paired Fisher’s exact test. Differences in the continuous variables comprised a non parametric paired Wilcoxon signed rank test to assess the changes between baseline values and all other time points within the groups (intragroup comparison). Differences between GnRHa treated and non treated patients (intergroup comparison) were assessed by the non-paired non parametric Mann–Whitney sign rank tests. Statistical significance was set at 5%. All statistical analyses were performed by the SPSS computer program, version 17.0 software for Windows (SPSS Inc., Chicago, IL, USA).

## Results

Baseline characteristics of CPP and EP girls are summarized in Table 1. All patients were in Tanner stage 2–3 of puberty. The CPP girls were younger (p = <0.001), had a higher BMI (1.1 ± 0.8 vs. 0.6 ± 0.7 yr, p = 0.004) and a higher prevalence of obesity and overweight (72.9 vs. 35.3%, p = 0.001) than the EP patients. Fasting glucose and lipids were in the normal range for age and sex according to Venezuelan reference values in all girls [26]. Mean total cholesterol levels at baseline, were however, 185.6 ± 35.7 mg/dl (in the 90^th^ percentile) in EP patients. The EP girls had higher fasting glucose (80.2 ± 10.2 vs. 73.3 ± 8.5 mg/dl, p = 0.04) and total cholesterol levels (185.6 ± 35.7 vs. 158.6 ± 31.9 mg/dl, p = 0.02) than CPP girls.

**Table 1:** Baseline characteristics of patients with CPP and EP

	**CPP**	**EP**	***p value***
n	37	34	-
Treated/Untreated	29/8	14/20	-
**Clinical characteristics**			-
Chronological age (yr)	7.4 ± 1.3	8.8 ± 0.6	**0.001**
Bone age (yr)	8.7 ± 2.1	9.3 ± 1.3	NS
Weight (Kg)	32.1 ± 6.7	32.6 ± 4.4	NS
Weight (z-score)	1.4 ± 0.8	1.9 ± 1.2	**0.001**
Height (SDS)	2.8 ± 1.2	1.3 ± 1.1	**0.001**
BMI (Kg/m2)	18.8 ± 2.4	18.2 ± 2	NS
BMI (z-score)	1.1 ± 0.8	0.6 ± 0.7	**0.004**
Obesity/Overweight n(%)	27 (72.9)	12 (35.3)	**0.001**
Obesity n(%)	9 (24.3)	3 (8.8)	NS
Overweight n(%)	18 (48.6)	9 (26.5)	0.054
Predict adult height (SDS) (SDS) (SDS)	0.3 ± 2.3	1.5 ± 1.2	**0.04**
Height velocity (SDS)	1.6 ± 2.1	1.8 ± 1.8	NS
**Hormonal evaluation**			
DHEAS (ng/ml)	51.1 ± 34.2	52.8 ± 23.7	NS
LH peak (IU/L)	10.9 ± 7.3	6.1 ± 4.5	**0.01**
LH to FSH stimulated ratio	1.1 ± 0.7	1.1 ± 1.7	NS
Estradiol (pg/ml)	22.3 ± 17	14 ± 11.8	**0.01**
Ovarian volume (ml)	1.7 ± 0.7	1.8 ± 0.5	NS
Uterine length (cm)	3.7 ± 1.2	3.4 ± 1	NS
**Metabolic profile**			
Fasting glucose (mg/dl)	73.3 ± 8.5	80.2 ± 10.2	**0.04**
Total Cholesterol (mg/dl)	158.6 ± 31.9	185.6 ± 35.7	**0.02**
LDL-C (mg/dl)	97 ± 35.1	113.2 ± 17.6	NS
HDL-C (mg/dl)	45 ± 8.9	53 ± 9.1	NS
TGR (mg/dl)	89.8 ± 46.7	104.8 ± 48.8	NS
TGR/HDL-C ratio	2.2 ± 0.9	1.5 ± 0.5	NS

Data are displayed as mean (± SDS). BMI, body mass index; DHEAS, dehydroepiandrosterone sulfate; LH, luteinizing hormone; FSH, follicle-stimulating hormone; LDL-C, low-density lipoprotein cholesterol; HDL-C, high-density lipoprotein cholesterol; TGR, Triglycerides.

Tables 2 and 3 describe clinical and laboratory characteristics of GnRHa treated and untreated females with CPP and EP, respectively, during 3 years of follow up.

**Table 2:** Three years evolution in GnRHa treated and untreated patients with CPP

	**Baseline**		**1 yr**		**2 yrs**		**3 yrs**		
	**Treated**	**Untreated**	**Treated**	**Untreated**	**Treated**	**Untreated**	**Treated**	**Untreated**	**After Tx**
**CA (yr)**	7.3 ± 1.5	7.7 ± 0.7	8.3 ± 1.5	8.7 ± 0.9	9.2 ± 1.5	9.6 ± 0.7	10.1 ± 1.5	10.7 ± 1.2	11.04 ± 0.4
**Weight (z-score)**	1.4 ± 0.7	1.2 ± 0.8	1.4 ± 0.6	1.3 ± 0.8	1.3 ± 0.9	1.2 ± 0.7	1.6 ± 0.6*	**0.9 ± 0.5**	0.8 ± 0.9
**BMI (z-score)**	1.2 ± 0.9	1 ± 0.8	1.2 ± 0.7	1.1 ± 0.8	1.1 ± 1	1 ± 0.7	1.3 ± 0.8	0.7 ± 0.5	0.7 ± 0.9
**Ob/over (%)**	79.3	50	65.4	50	65.4	50	81.8	40	40
**Obesity (%)**	24.1	25	26.9	25	23.1	25	45.5	0	20
**Overweight (%)**	55.2	25	38.5	25	42.3	25	36.4	40	20
**Glycemia (mg/dl)**	73.1 ± 9.1	74 ± 7.6	79.6 ± 8.9	88.6 ± 6.5	76 ± 10.1	84.3 ± 11.7	84.4 ± 5.3	80 ± 2.6	81 ± 18.4
**Total-C (mg/dl)**	156 ± 31.5	167.6 ± 35.7	172.1 ± 34.8	146.5 ± 42.3	152.8 ± 38.6	177.3 ± 23.9	159.2 ± 26.5	171.7 ± 39.1	167.3 ± 19.4
**LDL-C (mg/dl)**	96.6 ± 38.1	99.5 ± 16.3	108.1 ± 19.1	71.3 ± 24.1	92.9 ± 30.5	112.5 ± 7.8	100.2 ± 22.8	128.8 ± 13.9	122.5 ± 2.1
**HDL-C (mg/dl)**	43.8 ± 9	52 ± 7.1	35 ± 10.1	37.3 ± 9	48.5 ± 12.8	37 ± 4.2	43.8 ± 14	45.5 ± 2.1	44.5 ± 14.9
**TRG (mg/dl)**	83.3 ± 36.2	115.8 ± 78.5	80.9 ± 30.7	90.8 ± 33.7	79.9 ± 46.5	117 ± 66.8	83.3 ± 52.7	98.5 ± 37.5	64 ± 31.1
**TRG/HDL-C ratio**	2.1 ± 0.8	3 ± 1.5	2.5 ± 1	2.4 ± 0.5	1.7 ± 1.2	2.9 ± 2.2	1.9 ± 1.3	2.2 ± 0.9	2 ± 0.8

Data are expressed as mean ± SDS. SDS denotes the standard-deviation score for age and sex.

After Tx, after treatment; CA, Chronological age; BMI, body mass index; Ob/Over, obesity and overweight rate; Total-C, total cholesterol; LDL-C, Low density lipoprotein cholesterol; HDL-C, High density lipoprotein cholesterol; TGR, Triglycerides.

^*^p 0.02, treated vs. untreated at 3 years of follow up.

**Table 3:** Three years evolution of GnRHa treated and untreated patients with EP

	**Baseline**		**1 yr**		**2 yrs**		**3 yrs**		
	**Treated**	**Untreated**	**Treated**	**Untreated**	**Treated**	**Untreated**	**Treated**	**Untreated**	**After Tx**
**CA (yr)**	8.9 ± 0.6	8.8 ± 0.5	9.9 ± 0.6	9.8 ± 0.6	10.8 ± 0.7	10.7 ± 0.6	11.6 ± 0.7	11.5 ± 0.5	11.8 ± 0.4
**Weight (z-score)**	0.3 ± 0.7*	0.9 ± 0.6	0.1 ± 08*	0.9 ± 0.3	0.2 ± 0.9*	0.9 ± 0.4	0.1 ± 0.9*	0.9 ± 0.5**	-0.1 ± 1.1
**BMI (z-score)**	0.4 ± 0.7	0.9 ± 0.6	0.3 ± 0.8	0.8 ± 0.5	0.4 ± 0.9	0.7 ± 0.6	0.3 ± 1.2	0.8 ± 0.6	0 ± 0.8
**Ob/over (%)**	29	40	14	15	33	32	28.6	27	0
**Obesity (%)**	0	15	0	5	0	0	0	9	0
**Overweight (%)**	29	25	14	10	33	32	28.6	18	0
**Glycemia (mg/dl)**	79 ± 9.9	81.3 ± 11.1	89 ± 15.3	83.3 ± 9.7	84.3 ± 6.1	81.2 ± 8.6	84 ± 5.7	83 ± 6.4	83
**Total-C (mg/dl)**	195 ± 36.4	178.3 ± 35.5	184.8 ± 37.1	183.5 ± 33.8	186.8 ± 25	187.7 ± 41.8	133.5 ± 0.7	139.4 ± 37.6	123
**LDL-C (mg/dl)**	125 ± 38.4	113.2 ± 17.6	117.5 ± 42.8	131.5 ± 28.4	89.6 ± 66	105.8 ± 21.8	70.8 ± 5.1	83.7 ± 6.5	N.A
**HDL-C (mg/dl)**	50.6 ± 1.8	53 ± 9.1	51.3 ± 4.8	42.1 ± 7.4	47.7 ± 2.5	56 ± 24.3	49 ± 1.4	34.3 ± 4	N.A
**TGR (mg/dl)**	92.5 ± 30.9	113.4 ± 58.3	110 ± 43	73.9 ± 18	88 ± 18.5	85 ± 36	68.5 ± 14.9	96.5 ± 36.6	N.A.
**TGR/HDL-C ratio**	1.8 ± 0.6	1.5 ± 0.5	2.2 ± 1.3	1.9 ± 0.5	1.8 ± 0.3	1.5 ± 1	1.4 ± 0.3	2 ± 0.5	N.A.

Data are expressed as mean ± SDS. SDS denotes the standard-deviation score for age and sex.

After Tx, after treatment; CA, Chronological age; BMI, body mass index; Ob/Over, obesity and overweight rate; Total-C, total cholesterol; LDL-C, Low density lipoprotein cholesterol; HDL-C, High density lipoprotein cholesterol; TGR, Triglycerides.

*p <0.05 treated vs. untreated patients.

**p 0.03 untreated vs. patients after stop treatment.

### Central Precocious Puberty patients (CPP)

Weight and BMI z-scores remained unchanged over the first 2 years in both GnRHa-treated and untreated subjects. However, at 3 years of follow up, weight z-scores were higher in GnRHa-treated than in untreated patients (p = 0.02) and BMI z-scores followed this same trend, but did not reach significance (p = 0.06). A decreasing trend in weight and BMI z-scores was also detected in GnRHa-treated patients shortly after stopping therapy, but did not reach significance (n = 37, see Table 2).

At the time of diagnosis, both GnRHa-treated and untreated patients had a high prevalence of obesity and overweight. During 3 years of follow up, the prevalence of obesity and overweight remained unchanged in both groups of patients. In GnRHa treated patients who stopped treatment following at least 2 years of therapy, the rate of obesity and overweight showed a decreasing, although non significant trend (p = 0.2). Therefore, at 3 years of follow up the prevalence of obesity and overweight were similar between groups, but tended to be lower in untreated patients and in treated girls shortly after GnRHa discontinuation. Fasting glucose, total cholesterol, LDL, HDL-cholesterol, triglycerides and the triglyceride/HDL-cholesterol ratio, remained unchanged over the 3-year period, regardless of whether patients were treated or not. We found no differences between treated and untreated children for fasting glucose and lipids at baseline and during 3 years of follow up.

### Early Puberty patients (EP)

Weight and BMI z-scores remained unchanged over 3 years in both GnRHa-treated and untreated subjects. However, during the 3 year follow up period, weight z-scores were higher in untreated than in GnRHa-treated patients (p = 0.007, p = 0.002, p = 0.02, p = 0.04 at baseline, 1, 2 and 3 years, respectively); a similar trend was observed in BMI z-scores, but the differences were not significant. Weight z-scores were also significantly higher in untreated subjects than in treated subjects shortly after stopping therapy (p = 0.03) (n = 34, see Table 3).

Prevalence of obesity-overweight remained unchanged over 3 years of follow up regardless of whether patients were treated or not. A decreasing tendency was, however, noted in GnRHa-treated girls shortly after therapy discontinuation. Fasting glucose, total cholesterol, LDL, HDL-cholesterol, triglycerides and the triglyceride/HDL-cholesterol ratio, remained unchanged over the 3-year period, regardless of whether patients were treated or not. We found no differences between treated and untreated children for fasting glucose and lipids at baseline and during 3 years of follow up.

## Discussion

Our study confirms the high prevalence of obesity and overweight in girls with CPP and EP at the time of diagnosis. However, CPP girls presented with a higher BMI z-score and a higher prevalence of obesity and overweight than EP patients at baseline. The reason why so many subjects with CPP and EP have a BMI SDS above the 90^th^ percentile for chronological age is unclear. Whether the hormonal changes of puberty trigger an increase in BMI or whether a preexisting increased in BMI contributes to the onset of puberty at an earlier age is still not clear [29,30].

However, our study showed no difference in the BMI z-score and the obesity/overweight rates over the 3-year period of follow up, regardless of whether patients with CPP or EP were treated or not. This finding is confirmed by previous studies [1,14] that reported no significant change in BMI or obesity/overweight prevalence after GnRHa therapy in subjects with CPP or EP.

An interesting finding emerging at 3 years of follow from our study is the higher weight z-score noted in GnRHa treated than in untreated girls with CPP, with a similar trend being observed for the BMI z-score. This finding would suggest a negative influence of GnRHa treatment on body weight after 3 years of follow up in CPP girls. Furthermore, both our patients with CPP or EP showed a decreasing trend in BMI z-scores and in the obesity/overweight rate following GnRHa treatment discontinuation. Accordingly, some previous studies have demonstrated that the mean BMI z-score returned to pre-treatment values after therapy withdrawal [2,9,19].

The administration of GnRHa to girls with EP has been associated with greater BMI z-score at the end of therapy [19]. However in our cohort of EP patients, it is of interest to note that non-treated girls had a significantly higher weight z-scores than treated patients at baseline and that this difference remained unchanged over the three year follow-up, despite GnRHa treatment.

Sorensen et al. [20] observed higher fasting insulin, triglycerides, and LDL-cholesterol levels compared with controls in 23 girls with CPP and EP at the time of diagnosis. In our cohort of girls with CPP and EP, glucose, triglycerides, LDL- and HDL-cholesterol and the triglyceride/HDL-C ratio were in the normal range at the time of diagnosis and remained unchanged at 1, 2 and 3 years of follow up, regardless of whether they were treated or not. An unexpected finding are the higher fasting glucose and total cholesterol levels noted in EP than in CPP patients at the time of diagnosis, despite the higher BMI and obesity/overweight prevalence in the latter group. Even though we cannot explain this finding, it lead us to suggest that the higher prevalence of obesity/overweight in CPP patients could be an artifact due to the shift of the SD curve in puberty that is now applied on younger children.

A limitation of this study is the small sample of girls with CPP or EP, especially after therapy withdrawal, which could limit the statistical power of the analysis. As to insulin measurements, the blood sample we had was small and did not allow us to measure this parameter in our analysis. Data regarding the parental weight is lacking in different studies, including ours, and increased weight in parents certainly increases the risk of being overweight in childhood [31].

In summary, there is a high prevalence of obesity and overweight among girls with CPP and EP at diagnosis. This risk is greater in CPP than in EP girls. BMI z-scores and obesity/overweight rates remain stable during GnRHa therapy in girls with CPP and EP, but tend to decrease in GnRHa treated patients once therapy is discontinued for several months. Weight z-scores were higher at 3 years in GnRHa treated than in untreated girls with CPP, while they were higher in untreated subjects with EP during the whole three year follow up period. Fasting glucose and lipids, however, were in the normal reference ranges at the time of diagnosis and remained stable during the 3 year period of follow up, regardless of whether patients were treated or not, so that the metabolic profile of CPP and EP patients does not seem to be adversely affected by GnRHa therapy.

Therefore, physicians treating patients with CPP and EP must be aware of the increased BMI and obesity-overweight prevalence rates seen at the moment of diagnosis in these children and consider that these parameters could persist or even increase further during GnRHa treatment.

## Abbreviations

CPP: Central precocious puberty; EP: Early puberty; GnRHa: Gonadotropin releasing hormone analog; BMI: Body mass index.

## Competing interests

The authors declare that they have no competing interests.

## Authors’ contributions

AC participated in the design of the study, recollected the data, performed the statistical analysis and drafted the manuscript. RL conceived of the study, participated in the design and coordination of the study, contributed part of the study population and helped draft the manuscript. PG participated in the design of the study and contributed part of the study population. All authors read and approved the final manuscript.
